# Is It Possible to Reduce the Relative Age Effect through an Intervention on Motor Competence in Preschool Children?

**DOI:** 10.3390/children8050386

**Published:** 2021-05-13

**Authors:** Marcos Mecías-Calvo, Víctor Arufe-Giráldez, Miguel Cons-Ferreiro, Rubén Navarro-Patón

**Affiliations:** 1 Facultad de Ciencias de la Salud, Universidad Europea del Atlántico, 39011 Santander, Spain; marcos.mecias@uneatlantico.es; 2 Centro de Investigación y Tecnología Industrial de Cantabria (CITICAN), 39011 Santander, Spain; 3 Facultad de Ciencias de la Educación, Universidad de A Coruña, 15008 A Coruña, Spain; 4 Facultad de Formación del Profesorado, Universidade de Santiago de Compostela, 27001 Lugo, Spain; miguel.cons@usc.es (M.C.-F.); ruben.navarro.paton@usc.es (R.N.-P.)

**Keywords:** physical education, specific intervention, childhood, manual dexterity, aiming and catching, balance, MABC-2

## Abstract

The purpose of the study was to find out whether a short 6-week intervention on motor competence can reduce the Relative Age Effect (RAE) of preschool children born in the first quarter, compared to those born in the fourth quarter of the same year. Seventy-six preschool children (5.20 ± 0.54 years) from Lugo (Spain) participated. A quasi-experimental pre-post-test design was used with an intervention group (*n* = 32) and a control group (*n* = 44). The Movement Assessment Battery for Children-2 (MABC-2) was used to collect data before and after the intervention. The data show that, before the intervention, there are significant differences between the control and the intervention group in favor of the former (born in the first quarter of the year) in manual dexterity (*p =* 0.011), balance (*p =* 0.002), total test score (*p =* 0.008), and total percentile score (*p =* 0.010). After the application of the specific intervention, statistically significant differences were found in aiming and catching (*p <* 0.001), balance (*p =* 0.022), total test score (*p =* 0.001), and total percentile score (*p <* 0.001) in favor of the intervention group (born in the last quarter of the year). The results obtained suggest that the application of a specific intervention on MC could positively influence the improvement of MC in preschool children (boys and girls) and reduce the differences produced by the RAE.

## 1. Introduction

School physical education must provide all children with the acquisition of a sufficient motor competence (MC) to develop the psychomotor skills they need [1] to be able to access a wide range of physical-sport activities throughout their lives [2,3]. In this sense, preschool children during childhood go through a particularly sensitive and fundamental period for the development of their MC [4]. For this reason, MC is an important element of physical education (PE) curricula in many countries, including Spain [5,6]. The main objective of these school curricula is to develop the MC of all children, not only that of the most capable [7,8], because not all of them follow the same learning rhythm [9] and because it will be the basis for the development of more complex motor skills, typical of later educational stages (primary and secondary education) [4,10,11]. It is known that a poor MC in preschool children is associated with a lower participation in sports activities [12], a higher percentage of abandonment of sports practices [13], and a lower probability of being chosen in the detection of sports talents [14], among other factors.

The scientific literature shows ambiguous results regarding preschool children and MC, due to individual differences in their own development and maturation, depending on age, gender [15,16,17], and context [9]. On the other hand, recent studies indicate that these differences may be due to the relative age effect (RAE), highlighting that children who are born earlier within the same year and belong to the same class group obtain better results in MC than those who are born later [18,19,20,21]. Specifically in PE, where motor performance has an inverse relationship with age [22], this effect has important implications in terms of evaluation [23], since those born later obtain worse results [23,24,25] and have running, jumping, catching, or aiming difficulties [8], reinforcing the competence of older preschool children [12] and generating feelings of failure in those born later [26].

Regarding gender, the scientific literature indicates that girls perform better in manual dexterity [19,20,27] and balance [19,20,27,28,29] than boys. On the other hand, boys have higher levels of global MC, as well as better control and object manipulation skills [19,20,28,30].

Despite these negative consequences, a planned, progressive, and structured work should be provided in PE classes, based on quality and movement control [31], leading to an MC improvement [32], and later, expertly perform different motor skills (i.e., fine (writing or finger movements) and gross (throwing a ball or maintaining balance)) [9]. 

In this sense, MC interventions in preschool children are effective, whether proposed in the short or long term (4–8 weeks) or ≥6 months, respectively) [33,34,35,36,37,38]. Therefore, for preschool children to be physically competent and for the RAE to not exist, or be diminished, PE classes must be considered as privileged learning environments for the acquisition of the MC [39,40].

Taking into account that most PE classrooms present these circumstances and that interventions must be implemented to develop an adequate MC to reduce RAE [34,35], more research is necessary. Therefore, the aim of this study was to find out whether a short 6-week intervention on motor competence can reduce the RAE of preschool children born in the first quarter, compared to those born in the fourth quarter of the same year. To respond to this objective, the following research questions are posed:

(1) Are there differences in MC between preschool children born in the first and fourth quarter of the same year?
(2) In what motor skills are there these differences observed among preschool children?
(3) Are there differences in MC based on gender before the intervention? And after?
(4) Does an intervention on motor competence reduce the differences between preschool children born in the first and fourth quarter and, therefore, reduce the RAE?
(5) Are possible differences based on gender reduced once the intervention is performed?

## 2. Materials and Methods

### 2.1. Study Design

A quasi-experimental research with pre and post-test measures with a control group was conducted [41], where the dependent variables were the MABC-2 battery test (i.e., manual dexterity; aiming and catching; balance; total test score; total percentile score), and the independent variables were group (control group—CG: children born from January to March vs. intervention group—IG: children born from October to December) and gender (boys vs. girls).

The study was developed based on the requirements established in the Declaration of Helsinki for research with human participants, and was approved by the ethics committee of the Educa platform (code 22019).

### 2.2. Participants

Two urban educational centers in Lugo, Galicia (Spain), with a medium-high socioeconomic position, were invited to participate in the study. A total of 193 preschool-age children aged 4 to 5 years were invited, of which 20 were excluded for not correctly providing informed consent (signature or within the time established for it) and 92 for not having been born between January and March (Q1; quarter 1) or between October and December (Q4; quarter 4), and 5 more were excluded to obtain a final percentile below than 5. The final sample consisted of 76 preschool children. 

### 2.3. Measurements

The Spanish version of the MABC-2 battery was the assessment tool used [42]. It has proven to be a valid and reliable test to measure changes in MC over time [42,43,44] in children of different ages, with very high inter-rater reliability [45]. This tool consists of eight tests, lasting 20 to 40 min depending on age and difficulty experienced by the child, subdivided into three dimensions (three of manual dexterity (MD), two of aiming and catching (A&C), and three of balance (Bal), obtaining a scalar score in each test. The sum of all the scalar scores from each test provides a total score for each dimension. With these total scores, using a table of equivalences provided by the battery manual, the scalar score of the dimension is obtained, and through this, the percentile.

With the sum of the scalar scores of the eight tests, the total score of the battery was obtained, which, as in the case of the three dimensions, allows for the obtaining of the scalar score and the total percentile of the battery [43].

### 2.4. Procedures

Initially, the participation of the centers was requested, and once the proposal was accepted, the teachers were informed of the objectives and procedures of the study. Likewise, the legal guardians (i.e., parents) of the preschool children were also informed, and once the informed consent was signed and delivered, the MABC-2 battery was applied.

Evaluators were trained based on the battery instruction manual for the correct application of the MABC-2 and they always performed the same procedure to try to avoid bias: (1) task description; (2) examiner demonstration; (3) child practice following the procedure (evaluator could correct possible errors); (4) executing the test as indicated in the manual (no instructions were given during the test). The evaluators assessed the same class group at the beginning and at the end of the intervention and did not know which group each child belonged to.

Once the students from the two centers were evaluated, preschool children who were not born in the first quarter (born between 1 January and 31 March) or in the fourth quarter (born between 1 October and 31 December) were excluded.

For the IG (born in the fourth quarter), the intervention replaced PE classes by agreement with the management of the educational center since for this the sports facilities of the center (sports center) and the established schedules were used so as not to hinder the normal development of preschool classes. In this sense, a session was held each week, with a duration of 40 min, for 6 weeks (240 min) with the following structure: (1) warm-up or reception moment (5 min); (2) three-four tasks depending on the skill (MD, A&C, Bal; 30 min); (3) cool-down or goodbye moment (5 min). The distribution of the sessions was the same as that used by Navarro et al. [46,47] (Table 1), according to the objectives to be developed. 

The intervention was carried out in the sports center of the school where PE classes were taught, usually with conventional PE material (rings, Swedish bench, strings) available in schools and alternative material such as (tweezers, thread, beads, bottles). The teacher to student ratio was 15 preschool children, as in the CG. These sessions were given by a graduate in PE specialized in the training of children and PE teachers in infant and primary education. On the other hand, the PE teacher of each school continued with the planned planning without altering the program for the children in the CG (quarter 1), developing one of the aspects of the PE curriculum in pre-education in Spain (that is, the body and own body image, play and movement, daily activity, and personal care and health) [5]. The exact content was not recorded, but the duration or frequency of the procedure followed for the CG in each school was the same as in the IG, that is, 1 session a week of 40 min each session for 6 weeks. The teacher was unaware of the intervention that was carried out with the IG group and did not help in its application.

Once the process was completed, the MABC-2 battery was returned to both the CG and the IG the day after the intervention ended.

### 2.5. Statistical Analysis

For the statistical analysis, the IBM SPSS version 25 software (SPSS v.25, IBM Corporation, New York, NY, USA) was used. The level of significance was set at *p* < 0.05. The data were expressed in measures of central tendency (mean and standard deviation). First, to establish the equivalence between groups based on gender and age, the Chi-square test and an independent sample *t*-test were performed, respectively. Subsequently, to answer the research questions, the MABC-2 variables were analyzed before and after the intervention according to the group factor (CG-IG) and the gender factor (boy-girl) by means of a multivariate analysis of variance (MANOVA). The effect size was calculated using eta squared (η^2^) and the interaction between both factors (group and gender) was calculated using the Bonferroni statistic.

## 3. Results

Seventy-six healthy preschool children were evaluated of whom 26 (34.2%) were girls and 50 (65.8%) were boys aged 4 to 5 years old (mean = 5.15; SD = 0.56). The distribution of the participants was 44 preschool children from CG and 33 preschool children from IG.

All the variables of the MABC-2 battery (i.e., manual dexterity (*p* = 0.061), aiming and catching (*p* = 0.114), balance (*p* = 0.310), total test score (*p* = 0.675), and total percentile score (*p* = 0.578)) followed a normal distribution calculated with Levene’s test.

### 3.1. Baseline Characteristics

The initial characteristics of the participants regarding gender (*p* = 0.115) and age (*p* = 0.072) were similar in both groups (CG and IG). The baseline characteristics of the MABC-2 are outlined in Table 2.

The results of the multivariate analysis (MANOVA; Table 1) before the intervention, in terms of manual dexterity, indicate that there is a significant main effect in the type of group factor (F (1, 72) = 6.891, *p* = 0.011, η^2^ = 0.09), with the highest scores in the CG (born in the first quarter), but not in the gender factor (*p* = 0.510). Statistically significant differences were found in the interaction between both factors (F (1, 72) = 4.062, *p* = 0.048, η^2^ = 0.05). These differences occur between girls in both groups (*p* = 0.007), with the highest scores being in CG girls. Likewise, there are also differences between girls and boys in the CG (*p* = 0.029) with girls obtaining higher scores than boys.

In aiming and catching, no statistically significant differences were found in the group factor (*p* = 0.669), nor in gender (*p* = 0.784) or in the interaction between both factors (*p* = 0.700).

Regarding balance, the results indicate that there is a significant main effect with respect to the group factor (F (1, 72) = 5.620, *p* = 0.020, η^2^ = 0.07), being the factor with the highest scores in the CG group. A significant main effect was also found in the gender factor (F (1, 72) = 9.513, *p* = 0.003, η^2^ = 0.12), with higher scores in girls than in boys. No significant effect was found in the interaction between both factors.

In relation to the total test score, a significant main effect was found with respect to the group factor (F (1, 72) = 5.505, *p* = 0.008, η^2^ = 0.09), with the highest scores in the CG. A significant main effect was also found in the gender factor (F (1, 72) = 4.172, *p* = 0.047, η^2^ = 0.05), with higher scores in girls. No differences were found in the interaction of both factors (*p* = 0.596).

Finally, with respect to the total percentile score, only a statistically significant main effect was found in the group factor (F (1, 72) = 6.947, *p* = 0.010, η^2^ = 0.09), with the higher scores in CG.

### 3.2. Control vs. Intervention Group Outcomes after Intervention

The characteristics of MABC-2 after the intervention are outlined in Table 3 according to group and gender.

After the application of the intervention, the results of the multivariate analysis (MANOVA), in terms of manual dexterity, do not indicate statistically significant differences with respect to the group factor (*p* = 0.479) and, therefore, RAE was reduced. Differences were not found in the gender factor (*p* = 0.421) as had occurred before the intervention, nor in the interaction between both factors (*p* = 0.216), so the differences between girls in both groups disappear, as with between boys and girls in the control group.

Regarding aiming and catching, the results indicate that there is a significant main effect in the group factor (F (1, 72) = 18.676, *p* < 0.001, η^2^ = 0.21), with IG children obtaining higher scores, so the intervention program reduces RAE. A significant main effect was also found in the gender factor, which did not exist before the intervention (F (1, 72) = 4.073, *p* = 0.047, η^2^ = 0.05), with boys obtaining higher scores than girls. No statistically significant differences were found in the interaction of both factors (*p* = 0.792).

If we analyze the balance, the results indicate that there is a significant main effect in the group factor (F (1, 72) = 5.487, *p* = 0.022, η^2^ = 0.07), with the IG preschool children obtaining higher scores, therefore, the intervention program reduces the RAE with respect to this variable. A significant main effect was also found in the gender factor (F (1, 72) = 5.487, *p* = 0.022, η^2^ = 0.07), with girls obtaining higher scores than boys, as was the case before the intervention. No statistically significant differences were found in the interaction of both factors (*p* = 0.385).

Finally, if we analyze both the total test score and the total percentile score, a significant main effect was found in the group factor (F (1, 72) = 12.362, *p* = 0.001, η^2^ = 0.15) and (F (1, 72) = 13.439, *p* < 0.001, η^2^ = 0.16), respectively, with higher scores in the IG, so the intervention not only reduces RAE but also improves the overall scores in terms of motor competence. No differences were found regarding the gender factor or its interaction.

## 4. Discussion

To the authors’ knowledge, this is the first study in Spain that evaluates the effect of a specific intervention on MC and assesses the RAE reduction among preschool children born in the first and last quarter of the same year, based on gender. The results obtained in this research suggest that an intervention of this type reduces the RAE in preschool children of the same class with respect to MC and reduces the differences between boys and girls. This may be due to the fact that the intervention was carried out by a PE specialist in the IG, due to the pedagogical climate, the content, and the structure of the sessions, as occurs in other investigations [35,38,48,49], in comparison with other interventions that were developed by non-specialist PE teachers.

Before the intervention, the CG had significantly higher scores than the IG in manual dexterity, balance, total test score, and total percentile score. In aiming and catching, the scores were also slightly higher in the CG but were not statistically significant. Therefore, RAE exists in these dimensions as in previous studies [19,21]. These differences may be due to the physical size and maturation of those born earlier [50], which is sometimes confused with a higher capacity [51].

In terms of gender, before the intervention, there were differences between boys and girls in CG, with all scores being significantly higher in girls, except in aiming and catching, as in other studies [19,20,21,27,28,29,52,53,54]. In the IG, there were also no statistically significant differences between boys and girls in any of the variables studied. Scores on all tests are higher for girls than for boys, as in previous studies [19,20], except for manual dexterity. These differences could be due to the stereotyped activities, sporting or not, that each gender performs [53].

Once the intervention was performed, the scores of the different dimensions in the CG remained similar to those prior to the intervention, not following the pattern that would be expected due to maturation [15,16,17,55], although this intervention only lasted 6 weeks. In this group, the differences between boys and girls are maintained in all dimensions except in aiming and catching, where these differences become significant because boys obtain better scores [7,44,54,56,57,58,59].

After the intervention in the IG, the scores of the different dimensions increased significantly, so it can be said that the intervention program applied produces improvements in the MC [35,60,61,62,63] and, therefore, reduces the RAE with respect to the CG. Analyzing the pre-post-test differences according to gender, the scores in all variables regardless of gender increased both in boys and girls. However, statistically significant differences were only found in balance in favor of girls, that did not exist before [55,64,65], and in aiming and catching there are differences in favor of boys. In general, a structured program in MC can benefit both boys and girls [35,60,63].

Finally, if the IG and CG are compared once the intervention program was applied, statistically significant differences appeared [61] in aiming and catching, balance, total test score, and total percentile score, as in previous studies [35,60,61,62,63] in favor of the IG, so it can not only be indicated that RAE is reduced, but that the IG improves with respect to the CG. On the other hand, the differences in manual dexterity disappear. Although these types of activities are not directly related to school PE, it must be taken into account that their work produces these results. These results may indicate that, with a short intervention of 6 weeks and a session of 30 min per week, the RAE between the CG and the IG reduces, since the conditions of the preschool children remained stable (that is, context and individuals).

In addition to the contributions of our study, we must take into account some limitations that should make us view the results with caution. The sample size is relatively small compared to previous studies. Furthermore, the results were only analyzed immediately after the intervention, and the maintenance of these results in the medium or long-term has not been followed up. Personal, environmental, and MC-related factors that may affect performance, at any given time, were also not taken into account, but an attempt was made to reduce them.

## 5. Conclusions

The results obtained indicate that there is RAE in manual dexterity, balance, total test score, and total percentage scores in favor of preschool children born in the first quarter compared to those born in the last quarter. On the other hand, the results suggest that the application of a specific intervention on MC could positively affect its improvement in preschool children (boys and girls) and reduce the RAE differences. This type of intervention produces positive effects in preschool children (both boys and girls), reducing the differences with respect to boys and girls born in the first quarter and those that belong to the control group.

## 6. Practical Applications

Based on the results of this study, a rethink of the type of evaluation that is carried out in PE classes through standardized tests, which can favor those born in the first quarter and harm the youngest within the same cohort is encouraged. Furthermore, and taking into account that preschool children have unequal MC according to the tasks performed (manual dexterity, aiming and catching, and balance), we propose: (1) designing and implementing sessions in which the characteristics and individual motor skills levels of students are taken into account to individualize learning and include, for example, manual dexterity tasks, although they are not of the PE curriculum in Spain; (2) design and implement PE curricular tasks that are achievable by all (boys, girls, adults, and children) in such a way that they follow a logical and organized progression and that generate a challenge; (3) it is proposed to increase the time for free play and specific PE during the school day; (4) take advantage of other school environments, such as recess or breaks in the classroom to perform programs based on the improvement of MC, since short-term intervention programs such as that developed in this research can improve MC.

## Figures and Tables

**Table 1:** Objectives and tasks performed in each of the 6 sessions.

Session Number	Objectives
Session 1 “I explore my body”	Introduce manual dexterity, balance, and global throwing and catching skills through games
Session 2 “I develop my motor skills”	Improve fine motor and manual dexterity, jot down tasks, grasp and balance
Session 3 “The art of catching”	Develop manual dexterity with both hands and practice the tasks of catching and receiving various objects
Session 4 “Sharpen your aim”	Improve fine motor skills in both hands. Develop aim and precision when throwing objects
Session 5 “Circus tightrope walkers”	Work on manual dexterity and fine motor skills, develop static and dynamic balance
Session 6 “Motor circuits”	Remember the motor circuit, tasks, and games performed in previous sessions. Work with manual dexterity, aiming, grip, and balancing

Adapted from Navarro-Patón et al. [46,47].

**Table 2:** Results of MABC-2 test before intervention based on the type of group and gender.

		Total Sample		Control Group		Intervention Group	
		Mean	SD	Mean	SD	Mean	SD
Manual dexterity	boys	9.72	1.65	9.84	1.64	9.58	1.69
	girls	10.38	1.94	11.00	2.00	9.00	0.75
	Total	9.94	1.77	10.31	1.86	9.43	1.43
Aiming and catching	boys	7.40	3.15	7.38	3.31	7.41	3.04
	girls	7.07	2.31	6.88	2.76	7.50	0.53
	Total	7.28	2.88	7.18	3.07	7.43	2.63
Balance	boys	9.92	2.70	10.53	2.48	9.25	2.83
	girls	12.23	2.37	12.77	1.92	11.00	2.92
	Total	10.71	2.80	11.45	2.51	9.68	2.91
Total test score	boys	8.84	1.77	9.30	1.71	8.33	1.73
	girls	10.00	1.83	10.44	1.82	9.00	1.51
	Total	9.23	1.86	9.77	1.83	8.50	1.68
Total percentile score	boys	36.60	20.24	41.61	20.20	31.16	19.23
	girls	49.53	21.17	54.55	21.02	38.25	17.81
	Total	41.02	21.34	46.90	21.29	32.93	18.86

Note: SD = standard deviation.

**Table 3:** Results of MABC-2 test after intervention based on the type of group and gender.

		Total Sample		Control Group		Intervention Group	
		Mean	SD	Mean	SD	Mean	SD
Manual dexterity	boys	9.92	2.52	9.38	3.08	10.50	1.58
	girls	10.46	1.58	10.55	1.68	10.25	1.38
	Total	10.10	2.24	9.86	2.64	10.43	1.52
Aiming and catching	boys	9.28	2.92	8.07	2.09	10.58	3.16
	girls	7.53	2.24	6.66	1.94	9.50	1.60
	Total	8.68	2.81	7.50	2.12	10.31	2.86
Balance	boys	10.76	3.19	10.23	4.14	11.33	1.57
	girls	12.07	2.60	11.33	2.74	13.75	1.16
	Total	11.21	3.05	10.68	3.63	11.93	1.81
Total test score	boys	10.08	2.72	9.07	3.11	11.16	1.71
	girls	10.15	1.73	9.55	1.61	11.50	1.19
	Total	10.10	2.41	9.27	2.59	11.25	1.58
Total percentile score	boys	52.28	27.36	42.38	30.96	63.00	17.94
	girls	52.00	20.85	44.88	19.71	68.00	13.66
	Total	52.18	25.18	43.40	26.69	64.25	16.91

Note: SD = standard deviation.

## Data Availability

The data presented in this study are not available in accordance with Regulation (EU) of the European Parliament and of the Council 2016/679 of 27 April 2016 regarding the protection of natural persons with regard to the processing of personal data and the free circulation of these data (RGPD).
